# The potential of zooplankton in constraining chytrid epidemics in phytoplankton hosts

**DOI:** 10.1002/ecy.2900

**Published:** 2019-10-18

**Authors:** Thijs Frenken, Takeshi Miki, Maiko Kagami, Dedmer B. Van de Waal, Ellen Van Donk, Thomas Rohrlack, Alena S. Gsell

**Affiliations:** ^1^ Department of Aquatic Ecology Netherlands Institute of Ecology (NIOO‐KNAW) P.O. Box 50 Wageningen 6708 PB The Netherlands; ^2^ Department of Ecosystem Research Leibniz‐Institute of Freshwater Ecology and Inland Fisheries (IGB) Müggelseedamm 301 Berlin 12587 Germany; ^3^ Department of Environmental Solution Technology Faculty of Science and Technology Ryukoku University 1‐5 Yokotani, Seta Oe‐cho Otsu 520‐2194 Japan; ^4^ Institute of Oceanography National Taiwan University No. 1, Section 4, Roosevelt Road Taipei 107 Taiwan; ^5^ Graduate School of Environment and Information Sciences Yokohama National University 79‐7, Tokiwadai Hodogaya‐ku 240‐8501 Japan; ^6^ Institute of Environmental Biology University of Utrecht P.O. Box 80.056 Utrecht 3508 TB The Netherlands; ^7^ Faculty of Environmental Sciences and Natural Resource Management Norwegian University of Life Sciences P.O. Box 5003 Ås NO‐1432 Norway; ^8^ Present address: Great Lakes Institute for Environmental Research (GLIER) University of Windsor 401 Sunset Avenue Windsor Ontario N9B 3P4 Canada

**Keywords:** allometric relationship, cyanobacteria, density dependence, food‐chain model, rotifer, trophic cascade

## Abstract

Fungal diseases threaten natural and man‐made ecosystems. Chytridiomycota (chytrids) infect a wide host range, including phytoplankton species that form the basis of aquatic food webs and produce roughly half of Earth's oxygen. However, blooms of large or toxic phytoplankton form trophic bottlenecks, as they are inedible to zooplankton. Chytrids infecting inedible phytoplankton provide a trophic link to zooplankton by producing edible zoospores of high nutritional quality. By grazing chytrid zoospores, zooplankton may induce a trophic cascade, as a decreased zoospore density will reduce new infections. Conversely, fewer infections will not produce enough zoospores to sustain long‐term zooplankton growth and reproduction. This intricate balance between zoospore density necessary for zooplankton energetic demands (growth/survival), and the loss in new infections (and thus new zoospores) because of grazing was tested empirically. To this end, we exposed a cyanobacterial host (*Planktothrix rubescens*) infected by a chytrid (*Rizophydium megarrhizum*) to a grazer density gradient (the rotifer *Keratella* cf*. cochlearis*). Rotifers survived and reproduced on a zoospore diet, but the *Keratella* population growth was limited by the amount of zoospores provided by chytrid infections, resulting in a situation where zooplankton survived but were restricted in their ability to control disease in the cyanobacterial host. We subsequently developed and parameterized a dynamical food‐chain model using an allometric relationship for clearance rate to assess theoretically the potential of different‐sized zooplankton groups to restrict disease in phytoplankton hosts. Our model suggests that smaller‐sized zooplankton may have a high potential to reduce chytrid infections on inedible phytoplankton. Together, our results point out the complexity of three‐way interactions between hosts, parasites, and grazers and highlight that trophic cascades are not always sustainable and may depend on the grazer's energetic demand.

## Introduction

Fungal parasites are among the most virulent emerging diseases and are expected to threaten both natural and man‐made ecosystems (Fisher et al. 2012). One of the better investigated emerging fungal diseases is chytridiomycosis, caused by parasitic chytrids such as *Batrachochytrium dendrobatidis*, which has been linked to population declines and extinctions of many amphibian species (Berger et al. 1998, Daszak et al. 1999, Fisher et al. 2009). Besides amphibians, Chytridiomycota infect a wide range of eukaryotic as well as prokaryotic host species, including all phytoplankton groups (Sparrow 1960). Chytrids parasitizing phytoplankton can reach over 90% disease prevalence and thereby control population dynamics and seasonal succession of phytoplankton (Reynolds 1973, Van Donk and Ringelberg 1983, Van Donk 1989, Ibelings et al. 2004, Frenken et al. 2017a).

Many bloom‐forming phytoplankton species are large‐sized and filamentous; they may also be toxin producers, which makes them less suitable food for zooplankton (Lampert 1987, Turner and Tester 1997). Chytrids that infect these inedible phytoplankton species, however, make organic compounds available to zooplankton by producing edible transmission stages (zoospores). Moreover, chytrid zoospores can reach densities of over 1,000/mL and are of a high nutritional quality, as they contain essential fatty acids and sterols (Kagami et al. 2007b, 2014, Jobard et al. 2010, Gerphagnon et al. 2018). This chytrid‐induced trophic link has been conceptualized in the “mycoloop” (Kagami et al. 2007a, 2014). The mycoloop has been tested with various zooplankton species, including rotifers, cladocerans, and copepods, and feeding on zoospores has been shown to facilitate zooplankton survival or even allow population growth (Kagami et al. 2007b, 2011, Searle et al. 2013, Schmeller et al. 2014, Agha et al. 2016, Frenken et al. 2016, 2018).

Predator–parasite links are common in food webs (Johnson et al. 2010), with parasites serving as prey for predators. When predators consume free‐living stages of parasites, we would expect suppression of parasite epidemics. If the hosts of parasites are inedible or toxic to predators, however, predators would rely on parasites as sole diet for survival and strong suppression of parasite epidemics would not be an optimal strategy if this reduced the zoospore production below the food threshold necessary for predator survival. Empirical evidence for the effect of consumption of parasites by predators and thus increased protection of the host population from disease is rare. Thus far, it has only been shown in amphibian–chytrid–zooplankton systems where the presence of rotifers in isolated ponds was accompanied by reduced probability of chytrid infections of amphibians (Searle et al. 2013, Schmeller et al. 2014). Moreover, in a diatom–chytrid host–parasite system, grazing by *Daphnia galeata hyalina* reduced intensity but not the prevalence of infection in the host population (Kagami et al. 2004).

In order for grazers to survive and reproduce on a diet made up solely of zoospores, there is an intricate balance between the production of zoospores to fulfill the grazers’ nutritional demands (for growth or survival), and the loss in production of infections (and thus new zoospores) due to grazing. Therefore, if grazing pressure by the total zooplankton population is high, a trophic cascade may be triggered in which many zoospores will be removed, leading to reduced infection prevalence and increased host population growth. During a bloom of filamentous cyanobacteria, this cascade may lead to enhanced inedible cyanobacterial biomass together with reduced concentrations of edible zoospores, thus limiting food availability to zooplankton, resulting in zooplankton population decline. If grazing pressure by the total zooplankton population is low, the relative impact of grazing on the zoospore concentration will be low. This will lead to a situation with many zoospores and higher infection rates, and thus lower inedible phytoplankton biomass. In this case, food availability to zooplankton is higher, resulting in a population increase. It is unclear how the abovementioned feedback between density of zoospores and zooplankton population growth may affect phytoplankton infection prevalence.

Here, we experimentally tested whether grazing by zooplankton can affect a trophic cascade and reduce chytrid prevalence in a phytoplankton host, but also whether the feedback of zoospore density on grazer population growth affects the potential for grazers to control the infection prevalence in the host. We exposed cultures of a freshwater filamentous cyanobacterium (*Planktothrix rubescens*) infected by chytrids to a density gradient of zooplankton (*Keratella*). We developed a dynamical food‐chain model to test the maximum potential of *Keratella* to suppress the level of chytrid infection on an inedible phytoplankton host. We then extended this model to other zooplankton groups of different body sizes.

## Materials and Methods

### Test organisms and stock culture conditions

Origin and culturing of test organisms are described in our earlier experiments (Frenken et al. 2017b, 2018). In short, *Keratella* cf. *cochlearis* cultures were isolated during spring (April 2016) from a rotifer population sampled in a small shallow pond in Wageningen, the Netherlands (51°59′16.3″ N 5°40′06.0″ E) and were grown polyclonally in sterile 12‐well plates (VWR, Amsterdam, the Netherlands). Each well contained 15 randomly picked rotifers with 4 mL sterile WC medium, a standard culture medium for phytoplankton (Guillard and Lorenzen 1972). The rotifers were fed *ad libitum* with the green alga *Chlorella sorokiniana* (CCAP 211/8K) in stock culture. Both *Keratella* and *Chlorella* were grown at 16°C in a temperature‐ and light‐controlled incubator (Snijders Labs, Tilburg, the Netherlands), at a 14:10 light dark cycle with 10 μmol photons·m^−2^·s^−1^. The phytoplankton–chytrid system used in this experiment was the filamentous cyanobacterial host *Planktothrix rubescens* NIVA‐CYA97/1 with its fungal parasite, the chytrid Chy‐Lys2009 (Sønstebø and Rohrlack 2011, Rohrlack et al. 2013). *Planktothrix* and Chy‐Lys2009 cultures were grown in a temperature‐ and light‐controlled incubator (Snijders Labs, Tilburg, the Netherlands) at 5 μmol photons·m^−2^·s^−1^ in a 14:10 lightdark cycle, at 24 and 16°C, respectively. Every other week Chy‐Lys2009 cultures were fed with fresh *Planktothrix* cultures 1:1 (v/v). All *Planktothrix* and *Chlorella* cultures were grown as batches in 100‐mL Erlenmeyer flasks with 50 mL suspension and were diluted 1:10 (v/v) every other week using WC medium (Guillard and Lorenzen 1972). Phytoplankton and chytrid cultures used in this study were monoclonal but not axenic. Prior to the experiment, all cultures were acclimated to the experimental conditions.

### Experiment

The experimental design consisted of a total of eight treatments in quadruplicate (*n* = 4, 32 experimental units) in which *Planktothrix* (P) infected by chytrids (I) were cultured in the presence of four different densities of *Keratella* (K1–4; Table 1). Additionally, four types of control treatments were run to test for *Keratella* survival without food (K2, *Keratella* density 2), for *Keratella* survival on *Planktothrix* only (PK2, *Planktothrix* with *Keratella* density 2), and for *Planktothrix* growth without and with parasite exposure excluding *Keratella* (P and IP, respectively). Experiments were performed in sterile 500‐mL Erlenmeyer flasks with 240 mL culture suspension. All bottles were placed in a temperature‐ and light‐controlled incubator at 16°C and 10 μmol photons·m^−2^·s^−1^ with a 16:8 light dark cycle (Infors HT Multitron 2, Infors Benelux B.V., Doetinchem, the Netherlands). Each bottle was gently shaken once a day and moved to a new random location within the incubator. The experiment lasted 14 d, equaling roughly to one *Keratella* generation time, eight *Planktothrix* generation times, and five chytrid generation times (Frenken et al. 2017b, 2018).

**Table 1:** The experimental design consisting of a total of eight treatments (*n* = 4, 32 experimental units) in which *Planktothrix* infected by chytrids were cultured in the presence of four different densities of *Keratella* (0 = absence, 1 = presence, higher numbers indicate relative multiplicative inoculation densities). Treatment names are combined of I (Infection), P (*Planktothrix*), and K (*Keratella*)

Treatment	*Planktothrix*	Chytrids	*Keratella*
P	1	0	0
IP	1	1	0
IPK1	1	1	1
IPK2	1	1	2
IPK3	1	1	4
IPK4	1	1	8
PK2	1	0	2
K2	0	0	2

To maintain a uniform age distribution in the *Keratella* populations, 3 days before the start of the experiment (*t*_−3_), all individuals (~5,300) were collected and mixed in an Erlenmeyer flask and distributed into two 12‐well plates. Two days before the experiment (*t*_−2_), all individuals were mixed again and washed five times in sterile WC medium to remove *Chlorella*. Subsequently, all *Keratella* were inoculated again in sterile WC medium and left to starve overnight to reduce the risk of transferring any *Chlorella* cells into the experiment. One day before the experiment (*t*_−1_), all individuals were mixed again and washed once more in sterile WC medium.

**Figure 1:**
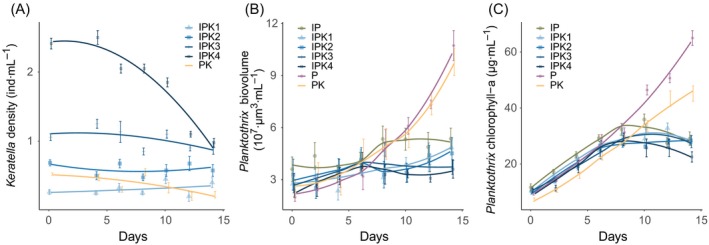
*Keratella* density in the treatments fed with uninfected (PK; orange line) or infected *Planktothrix* (A), total *Planktothrix* biovolume (B), and chlorophyll‐a concentration (C) in all treatments. Treatment names are combinations of I (Infection), P (*Planktothrix*), and K (*Keratella*); numbers for the IPK treatments indicate the grazer density level from lowest (1) to highest (4). Lines are Loess‐smoothed conditional averages; error bars denote mean ± SE (*n* = 4).

At the start of experiment (*t*_0_) *Planktothrix* and infected *Planktothrix* cultures were added to the experimental units and diluted to a total biovolume of 1 × 10^7^ μm^3^·mL^−1^ and a starting infection prevalence estimated at 12.5%. Prior to addition of *Keratella*, all prepared *Keratella* individuals were collected in one bottle and gently mixed to homogenize grazer population, from which different volumes subsequently were diluted in order to achieve the different grazing treatments, that is, grazing intensity gradient. The highest grazing density (K4) was set at 2.5 individuals/mL, which was subsequently diluted twice (K3 ~1.25 individuals·mL^−1^), four times (K2 ~0.63 individuals·mL^−1^) and eight times (K1 ~0.31 individuals·mL^−1^). Relative inoculation densities can also be found in Table 1, and achieved grazer densities are reported in Figure 1a.

### Measurements

From each bottle, 20 mL was sampled every other day for *Keratella* abundance, *Planktothrix* biomass and chl‐a concentration, zoospore density, and infection prevalence. Part of this sample was preserved immediately using 25% glutaraldehyde (Merck, Darmstadt, Germany) to a final concentration of 0.5% (v/v). As the *Planktothrix* and chytrid cultures were not axenic, we also assessed the growth of heterotrophic bacteria over time, because they can serve as a potential food source to *Keratella*. Therefore, a subsample of 2 mL was flash‐frozen in liquid nitrogen and stored at −80°C for quantification of heterotrophic bacteria using a flow cytometer. *Keratella* counts were performed on 10‐mL life samples using a stereomicroscope (LeicaWILD MZ8, Leica Microsystems B.V., Son, the Netherlands). No data are available for *Keratella* counts on day 2, and in subsequent calculations, *Keratella* density was therefore assumed to be the average of days 0 and 4. *Planktothrix* total biomass was determined in triplicate on a CASY Cell Counter (Schärfe System GmbH, Reutlingen, Germany). *Planktothrix* chlorophyll‐a concentration was estimated by fluorescence on a Phyto‐PAM with an Optical Unit ED‐101US/MP using the sum of the chlorophyll‐a signal (Heinz Walz GmbH, Effeltrich, Germany). For zoospore density, at least 250 cells were counted, or a minimum of 20 fields of view.

During normal growth conditions, the chytrid Chy‐Lys2009 only infects the end of host filaments. Infections were thus counted as a categorical variable: at least 100 ends of filaments were inspected in each sample, which were either infected (*i*) or uninfected (*ui*). The prevalence of infection was subsequently calculated as $P=i/\left(i+ui\right)$. All infection‐ related counts were performed at a magnification of 400× on an inverted microscope (DMI 4000B, Leica Microsystems CMS GmbH, Mannheim, Germany).

Heterotrophic bacteria were counted by flow cytometry based on the protocol of Brussaard (2004). To this end, bacterial samples were thawed just before analysis and diluted in a Tris‐EDTA buffer (pH 8.2) to achieve an event rate between 100 and 1,000 events/s and stained with the DNA stain SYBR® Green I nucleic acid gel stain (Molecular Probes, Invitrogen, Paisley, United Kingdom) for 15 min in the dark at room temperature (final concentration of 5 × 10^−5^ of commercial stock). These were analyzed by an Influx Cell Sorter (BD Biosciences, Franklin Lakes, New Jersey, USA) equipped with a 200‐mW 488‐nm laser (Coherent Inc., Santa Clara, USA). Trigger was set to 530/40 (488 nm) at a level of 0.7. Measurement was corrected with blanks, consisting of stained Tris‐EDTA buffer. The bacteria were gated in a plot of side scatter vs. 580/30‐nm excitation and processed in the BD FACS Sortware software. The analyzed volume was calculated by weighing the sample before and after analysis.

### Data analysis

As zoospore counts reflected the standing stock of zoospores at a given time point (i.e., the total production minus all losses) and therefore implicitly already included the effect of grazing, we estimated relative food availability (zoospores *Keratella* per individual [ind]) by dividing the cumulative amount of zoospores removed (zoospores/mL) over the cumulative *Keratella* abundance (ind/mL). Zoospore removal was calculated by subtracting the realized zoospore concentration in each grazed bottle by the zoospore concentration in the infected control without grazer. Ingestion rates (ng C·ind^−^·h^−1^) were estimated by multiplying zoospore removal per *Keratella* with mean zoospore carbon contents published earlier using the same host–parasite system under the same environmental conditions (Frenken et al. 2017b).

To test if the time courses of each measured variable differed between treatments, a repeated‐measurements ANOVA (RM ANOVA) was performed in the statistical package Statistica 13.2 (Statsoft Europe, Hamburg, Germany). Variables were tested for normality and equal variance using the Shapiro‐Wilk and Levene's tests, respectively. Relative food availability was square‐root transformed to improve normality.

Treatment effects on average ingestion rates were analyzed using a one‐way ANOVA in SigmaPlot for the time course of high food availability (days 6–10) and averaged over the entire experiment period. Variables were tested for normality and equal variance using the Shapiro‐Wilk and Brown‐Forsythe test, respectively. Pairwise comparisons were conducted using the Tukey post hoc test.

### Model formulation, basic calculation and parameter estimation

#### Dynamical food‐chain model

Similar to other food‐chain models including parasitic fungi (Miki et al. 2011, Gerla et al. 2013, Almocera et al. 2018), we set *A*_*U*_, *A*_*I*_, *F*,* R* as uninfected *Planktothrix* (10^7^ μm^3^/mL), infected *Planktothrix* (10^7^ μm^3^/mL), free‐living zoospores of chytrids (cells/mL), and *Keratella* (ind/mL), respectively.

We assumed that uninfected *Planktothrix* show logistic growth but decrease at a simple mass action rate due to new infection (β*A*_*U*_ *F*) and with a density‐independent mortality rate *m*_*A*_ (per day). The equation is given as

(1) $$\frac{dA_{U}}{\mathrm{dt}}=r_{A}\left(1-\frac{A_{U}}{K}\right)A_{U}-\beta A_{U}F-m_{A}A_{U},$$

where *r*_*A*_, *K*, β represent the intrinsic growth rate (per day), carrying capacity for the uninfected host cells (10^7^ μm^3^/mL), and infectivity of chytrids (mL/cell), respectively.

The abundance of infected *Planktothrix* increases with new infection but decreases with maturation of chytrids sporangia with the development time τ (d) and density‐independent mortality with a rate *m*_*A*_ (per day). The dynamic is given by

(2) $$\frac{dA_{I}}{\mathrm{dt}}=\beta A_{U}F-\left(\frac{1}{\tau }\right)A_{I}-m_{A}A_{I}.$$

The abundance of chytrid zoospores increases with the development of sporangia and release of new zoospores. The number of newly produced zoospores per infected host biomass is set as *b*. The loss of zoospores is assumed to occur when attaching to the host for infection. In addition, the zoospores decrease with grazing by the rotifer with a clearance rate *g*_*F*_ (mL/cell) and with the density‐independent mortality at a rate *m*_*F*_ (per day). The equation is given by

(3) $$\frac{\mathrm{dF}}{\mathrm{dt}}=\left(\frac{b}{\tau }\right)A_{I}-\beta A_{U}F-g_{F}RF-m_{F}F.$$

vThe abundance *R* of *Keratella* includes background growth with a rate *g*_*B*_ (per day; e.g., due to grazing on bacteria), growth by grazing on fungal zoospores at a clearance rate *g*_*F*_ (mL/ind) with the conversion factor *c*_*R*_ (ind per cell) (i.e., the ratio of the number of newly produced rotifer to the number of zoospores ingested), and density‐independent mortality at a rate *m*_*R*_ (per day) as follows:

(4) $$\frac{\mathrm{dR}}{\mathrm{dt}}=\left(g_{B}+c_{R}g_{F}F-m_{R}\right)R.$$

### Minimum prevalence of infection

The index for the prevalence of infection can be derived from Eqs. (2) and (4). At equilibrium, the abundance of infected hosts (*A*_I_*) can be calculated by setting *dA*_*I*_/*dt *=* *0 (Eq. (2)). Then, the equilibrium prevalence of infection *P* (F*) (=*A*_I_*/ [*A*_U_*+*A*_I_*]) can be calculated without using Eq. (1) as the function of the equilibrium density of chytrids (F*),

(5) $$P\left(F^{*}\right)=\frac{\beta F^{*}}{\beta F^{*}+m_{A}+\frac{1}{\tau }}.$$

We can further calculate the minimum abundance of zoospores that is required for the maintenance of the population of *Keratella*. The zoospore abundance should satisfy the following inequality to realize *dR*/*dt *>* *0 (Eq. (4)) with the assumption *m*_*R*_ – *g*_*B*_ > 0,

(6) $$F>\frac{m_{R}-g_{B}}{c_{R}g_{F}}\equiv F_{\min}.$$

Therefore, with the presence of *Keratella*, the zoospore abundance cannot be less than *F*_min_. Because the prevalence of infection at equilibrium (Eq. (5)) increases with the zoospore abundance (F*), we calculate *P*(*F*_min_) as *the minimum prevalence of the infection P*_min_; that is,

(7) $$P_{\min}=\frac{\beta F_{\min}}{\beta F_{\min}+m_{A}+\frac{1}{\tau }}.$$

This implies that the *Keratella* population is sustainably able to suppress the chytrid infection to *P*_min_. When the *Keratella* population suppresses the chytrid infection further, densities of chytrids will become too low to sustain *Keratella* population growth. When suppression is insufficient, chytrid infections will decimate the *Planktothrix* population entirely, leading to loss of chytrid zoospores and therefore insufficient food to sustain *Keratella* growth. In order to estimate this minimum prevalence of the infection *P*_min_, we estimated *m*_*R*_, *m*_*R*_ – *g*_*B*_, *c*_*R*_ *g*_*F*_, β, and *m*_*A*_ + 1/τ based on the results from the laboratory experiments. For the calculation steps, see supporting information.

The dynamical food‐chain model was also used to estimate the maximum potential of other zooplankton groups to suppress the level of chytrid infection by combining parameters derived from this culture experiment with literature values on the zooplankton size–clearance rate relationship. Clearance rates and body size measurements used in the model, and references to the publication they were retrieved from, can be found in Appendix S1: Table S1. We performed an extensive literature search in Google Scholar using a combination of search terms for common zooplankton species names (as in Fig. 5) with the terms “clearance rate” OR “ingestion rate,” including primary literature found in references of papers. Only species for which we could find high‐quality data for both clearance rates and body size measurements were used.

## Results

*Keratella* populations showed different growth dynamics in different treatments (Fig. 1a). In the treatment without any food (K2), *Keratella* died within 6 d (not shown in graph). In the treatments with *Planktothrix* but without infections (PK2) *Keratella* died off more slowly (Table 2, *F*_1,6 _= 133.1, *P* < 0.001). Addition of chytrids had a significant effect on *Keratella* population development (*F*_1,6 _= 119.5, *P* < 0.001), which generally showed a converging pattern across treatments (Fig. 1a). Specifically, the *Keratella* population in the highest grazing treatment (IPK4) decreased over time, whilst the populations in treatments IPK2 and IPK3 remained relatively stable and the populations in treatment IPK1 even grew slightly.

**Table 2:** Output of the RM ANOVA reporting significance, degrees of freedom, and the *F*‐value of treatment effect on different variables. Values in bold denote a significant effect (α < 0.05)

Effect	Variable		df	df_ERROR_	*F*	p
*Planktothrix*	*Keratella* abundance (ind·mL^−1^)	Treatment	1	6	133.1	**<0.001**
		Time	7	42	46.6	**<0.001**
		Time * Treatment	7	42	19.4	**<0.001**
Infection	*Keratella* abundance (ind·mL^−1^)	Treatment	1	6	119.5	**<0.001**
		Time	7	42	2.2	0.057
		Time * Treatment	7	42	4.7	**<0.001**
	Biovolume concentration (×10^7^ μm^3^·mL^−1^)	Treatment	1	6	1.2	0.316
		Time	7	42	19.5	**<0.001**
		Time * Treatment	7	42	8.3	**<0.001**
	Chlorophyll‐a concentration (μg·mL^−1^)	Treatment	1	6	90.1	**<0.001**
		Time	7	42	125.0	**<0.001**
		Time * Treatment	7	42	40.8	**<0.001**
	Bacteria density (×10^6^·mL^−1^)	Treatment	1	6	4.4	0.080
		Time	7	42	185.5	**<0.001**
		Time * Treatment	7	42	0.4	0.873
*Keratella*	Biovolume concentration (×10^7^ μm^3^·mL^−1^)	Treatment	1	6	0.0	0.990
		Time	7	42	45.4	**<0.001**
		Time * Treatment	7	42	0.9	0.550
	Chlorophyll‐a concentration (μg·mL^−1^)	Treatment	1	6	58.6	**<0.001**
		Time	7	42	201.5	**<0.001**
		Time * Treatment	7	42	6.5	**<0.001**
	Zoospore density (counts·mL^−1^)	Treatment	4	14	7.5	**0.002**
		Time	7	98	99.9	**<0.001**
		Time * Treatment	28	98	4.8	**<0.001**
	Bacteria density (×10^6^·mL^−1^)	Treatment	1	5	0.6	0.476
		Time	7	35	418.7	**<0.001**
		Time * Treatment	7	35	3.4	**0.007**
Grazing intensity	Biovolume concentration (×10^7^ μm^3^·mL^−1^)	Treatment	3	12	0.6	0.616
		Time	7	84	4.3	**<0.001**
		Time * Treatment	21	84	0.8	0.722
	Chlorophyll‐a concentration (μg·L^−1^)	Treatment	3	12	2.6	0.097
		Time	7	84	73.9	**<0.001**
		Time * Treatment	21	84	1.2	0.312
	Prevalence of infection (%)	Treatment	3	10	0.8	0.539
		Time	7	70	337.4	**<0.001**
		Time * Treatment	21	70	0.4	0.983
	Relative food availability (number of zoospores per *Keratella*)	Treatment	3	12	62.8	**<0.001**
		Time	7	84	43.4	**<0.001**
		Time * Treatment	21	84	2.6	**<0.001**
	Cumulative zoospores removed (counts·mL^−1^)	Treatment	3	12	3.5	**0.050**
		Time	7	84	158.0	**<0.001**
		Time * Treatment	21	84	2.9	**<0.001**
	Bacteria density (×10^6^·mL^−1^)	Treatment	3	8	0.6	0.632
		Time	7	56	323.9	**<0.001**
		Time * Treatment	21	56	1.2	0.254

*Planktothrix* total biovolume and chlorophyll‐a increased over time in all treatments but leveled off earlier in the chytrid infected treatments (Fig. 1b, c). Infections had no observable effect on total *Planktothrix* biovolume (*F*_1,6 _= 1.2, *P* = 0.316) but led to a lowered chlorophyll‐a concentration (*F*_1,6 _= 90.1, *P* < 0.001). Similarly, *Keratella* presence had no effect on total biovolume (*F*_1,6 _= 0.0, *P* = 0.990), but also led to a lowered chlorophyll‐a concentration (*F*_1,6 _= 58.6, *P* < 0.001). Different grazing intensities did not lead to significant changes in total biovolume and chlorophyll‐a concentration (*F*_3,12 _= 0.6, *P* = 0.616; *F*_3,12 _= 2.6, *P* = 0.097).

**Figure 2:**
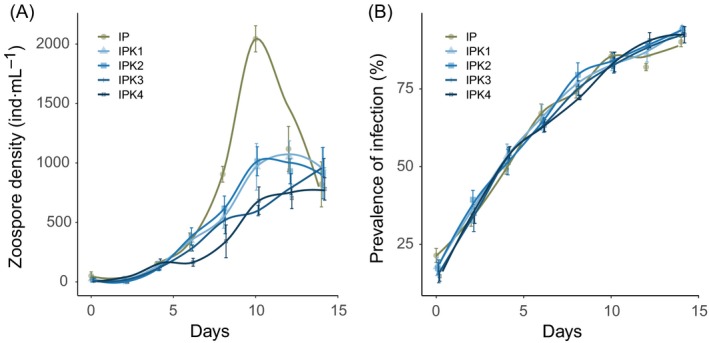
Zoospore density (A) and prevalence of infection (B) in all infected treatments. Treatment names are combinations of I (Infection), P (*Planktothrix*), and K (*Keratella*); numbers for the IPK treatments indicate the grazer density level from lowest (1) to highest (4). Lines are Loess‐smoothed conditional averages; error bars denote mean ± SE (*n* = 4).

Chytrid zoospore density showed a strong exponential increase in the treatment without grazing (Fig. 2a), which peaked around day 10 and afterwards decreased. In the grazed treatments zoospore density was generally lower (*F*_4,14 _= 7.5, *P* < 0.001). After 14 d of chytrid exposure, prevalence of infection increased from about 20 to 90% and displayed logistic growth (Fig. 2b), which was not significantly different between treatments (*F*_3,10 _= 0.8, *P* = 0.539).

**Figure 3:**
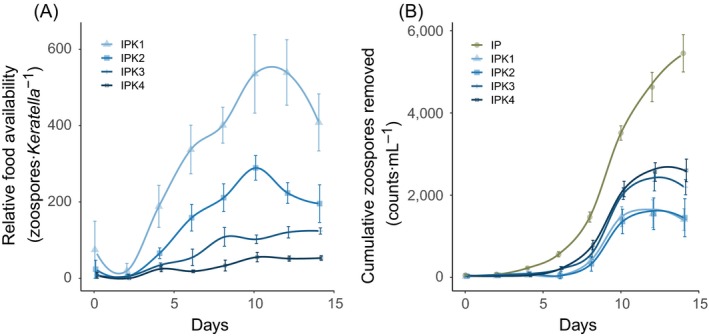
Relative food availability (A) and cumulative amount of zoospores removed as compared to the control (B). Values denote mean ± SE (*n* = 4). Treatment names are combined of I (Infection), P (*Planktothrix*), and K (*Keratella*); numbers indicate the grazer density level from lowest (1) to highest (4). Lines are Loess‐smoothed conditional averages; error bars denote mean ± SE (*n* = 4).

Relative food availability (Fig. 3a) was highest in the treatment with the lowest amount of grazers (IPK1) and generally decreased with *Keratella* density (*F*_3,12 _= 62.8, *P* < 0.001). Relative food availability was lowest in treatment IPK4 and reached a maximum of only 55 zoospores *Keratella* per ind, whilst it was a factor of 10 higher in the treatment with the lowest *Keratella* density.

The cumulative amount of zoospores produced in the control treatment (IP) reached about 5,500 zoospores·mL^−1^ after 14 d (Fig. 3b). Between 1,400 and 2,600 of the total 5,500 zoospores·mL^−1^ produced were grazed by the end of the experiment, which represented a 26–47% removal. Ingestion rates over the period of food repletion (day 6–10) ranged between 23 and 93 ng C·ind^−1^·h^−1^ (Table 3) and were the highest in IPK1 and lowest in IPK4. The average ingestion rate over the entire experiment did not differ markedly from that in the period of food repletion and ranged between 26 and 88 ng C·ind^−1^·h^−1^, being again higher in IPK1 as compared to IPK3 and IPK4.

**Table 3:** Ingestion rates of *Keratella* in the different treatments between days 6 and 10 (= period of food repletion), and average over the entire experiment (ng C·ind^−^·h^−1^). Values denote mean ± SE (*n* = 4). Superscripts represent output of pairwise comparison. Treatment names are combined of I (Infection), P (*Planktothrix*), and K (*Keratella*)

Treatment	d6–d10	Average
IPK1	93 ± 15^a^	88 ± 15^a^
IPK2	50 ± 13^ab^	45 ± 14^ab^
IPK3	54 ± 8^ab^	40 ± 3^b^
IPK4	23 ± 2^b^	26 ± 2^b^

**Figure 4:**
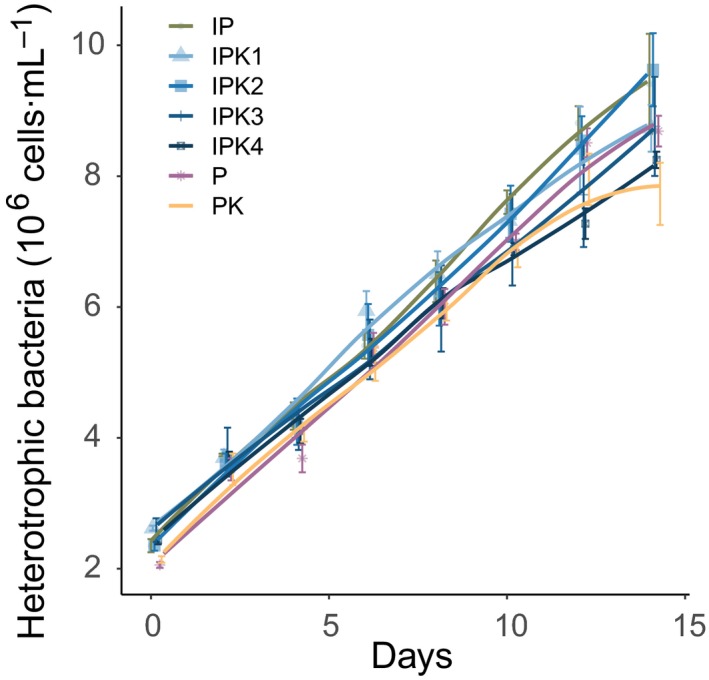
Bacteria densities in all treatments. Values denote mean ± SE (*n* = 4). Treatment names are combined of I (Infection), P (*Planktothrix*), and K (*Keratella*); numbers indicate the grazer density level from lowest (1) to highest (4). Lines are Loess‐smoothed conditional averages; error bars denote mean ± SE (*n* = 4).

Heterotrophic bacteria densities generally increased with a factor 4–5 over the course of the experiment, but were not significantly affected by infections (*F*_1,6 _= 4.4, *P* = 0.080), *Keratella* presence (*F*_1,5 _= 0.6, *P* = 0.476), or *Keratella* grazing density (*F*_3,8 _= 0.6, *P* = 0.632) (Fig. 4).

The model parameters were estimated by fitting model equations into experimental time series as (*m*_*R*_, *m*_*R*_ − *g*_*B*_, β, *m*_*A*_ + 1/τ, *c*_*R*_ *g*_*F*_) = (0.2338/d, 0.07460/d, 5.419 × 10^−4^ mL·ind^−1^·d^−1^, 0.05534/d, 1.561 × 10^−4^ mL·ind^−1^·d^−1^; see Appendix S1: Fig. S1). Then, using the estimated values of *m*_*R*_ *− g*_*B*_ and *c*_*R*_ *g*_*F*_ with Eq. (6), the minimum level of chytrid zoospores was obtained as *F*_min_ = 478 ind/mL. Substituting these estimated values of β, *m*_*A*_ + 1/τ, and *F*_min_ into Eq. (7), the minimum to which *Keratella* should suppress prevalence of infection (*P*_min_) to maintain a sustainable population was estimated as 82% in this experiment.

**Figure 5:**
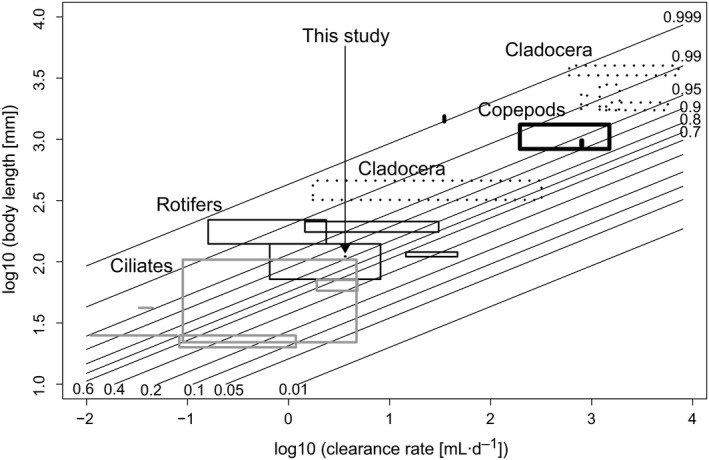
Minimum infection prevalence (fraction) to which the different zooplankton groups can potentially suppress the prevalence of chytrid infections while maintaining a stable population, as a function of zooplankton size and clearance rate. Rectangular boxes and lines indicate literature values for different zooplankton species.

Based on published values for body sizes and clearance rates (see Appendix S1: Table S1) we also estimated the minimal level to which other zooplankton groups could theoretically suppress chytrid infection, while still maintaining their population density (Fig. 5). Thus, the current model parameterized by *Keratella* was used to extrapolate to other zooplankton groups. Based on this model, copepods and Cladocera can suppress infection to a prevalence of infection of about 85 or 60%, respectively. Smaller‐sized zooplankton with lower clearance rates, such as rotifers and ciliates, show a higher removal potential; theoretically they can reduce infection to a minimum of roughly 25 and 10%.

## Discussion

We hypothesized that increasing zooplankton grazing intensity will lower chytrid zoospore density, resulting in a decreased prevalence of infection. Our data indeed show that grazing by zooplankton can reduce the amount of chytrid zoospores, but has no observable cascading effect on the infection prevalence and thereby *Planktothrix* population densities. Nevertheless, this work supports earlier findings (Frenken et al. 2018) showing that *Keratella* population growth can be sustained by a diet of zoospores during blooms of inedible filamentous cyanobacteria (i.e., mycoloop). However, *Keratella* are not able to protect the host from disease in our experimental setup. Our dynamical food‐chain model confirmed that *Keratella* cannot substantially reduce the prevalence of infection, but estimates based on body size–clearance rate relationships show that other zooplankton species might be able to reduce prevalence of infection to a lower level and simultaneously sustain population growth.

As was shown before, *Keratella* fed with *Planktothrix* infected by chytrids can maintain a net population growth rate that is comparable to that observed for populations grown on a green algal diet, and *Keratella* fed with uninfected *Planktothrix* did not survive (Frenken et al. 2018). Indeed, in the treatment without chytrids, *Keratella* abundance slowly decreased, likely because *Planktothrix* is not a good food source in terms of size, morphology, and nutritional value. Although there were significant amounts of bacteria present in the cultures, our data show no signs of an effect of *Keratella* grazing on bacteria. But even if *Keratella* did graze on bacteria, these may not represent a high‐quality food source needed for zooplankton to reproduce, probably because bacteria lack essential sterols (Martin‐Creuzburg et al. 2011, Freese and Martin‐Creuzburg 2013).

*Keratella* population development in the treatments with chytrids converged over time (Fig. 1). In the treatment with the lowest grazing density, populations could grow (IPK1), probably because of a higher relative food availability, which could support higher ingestion rates. In the treatments with a higher grazer density, fewer zoospores were available per capita of grazer, which led to reduced ingestion rates and as such could support less growth and led to relatively stable (IPK2, IPK3) or decreasing (IPK4) *Keratella* population densities. Because our experiment started with equal host densities, infection prevalence developed similarly in all infected treatments (Fig. 1b), which likely resulted in an equal net zoospore production rate in all infection treatments. Consequently, it is not surprising that the rotifer population density converged toward an intermediate plateau with an identical density in all treatments, resulting in a net increase or decrease of population densities depending on the starting population density.

*Keratella* grazing led to a 26–47% removal of total zoospores produced. Even though it seems a very significant reduction, this was not enough to constrain the amount of infections. The lack of grazing effect on the infection prevalence may be because of overproduction of zoospores by the chytrid, which may be an adaptive response to compensate potentially high mortality in zoospores associated with, for instance, predation, natural death, adsorption to suspended matter, or a stressful environment. If higher rotifer densities had been applied at the start of the experiment, possibly most of the zoospores would have been grazed away early in the experiment, subsequently resulting in fewer infections later in the experiment. However, a rotifer population with higher densities would likely have shown similar patterns as observed for treatment IPK4, converging toward a lower plateau at which a rotifer population can actually be sustained by the realized zoospore production.

The average ingestion rate by *Keratella* over the entire experiment was estimated to range between 26 and 88 ng C·ind^−1^·h^−1^ in IPK4 and IPK1, respectively. Our results indicated that an ingestion rate in the range of 20–40 ng C·ind^−1^·h^−1^ was too low to facilitate *Keratella* survival, an ingestion rate in the range of 40–50 ng C·ind^−1^·h^−1^ could support a stable population of *Keratella* to survive, and ingestion rates above 80 ng C·ind^−1^·h^−1^ supported reproduction and thereby population growth. Carbon ingestion rates on a diet of zoospores are rarely quantified. Earlier work by Kagami et al. (2017) estimated that ingestion rates of Cladocera feeding on zoosporic carbon derived from indigestible pine pollen roughly ranges between 70 and 180 ng C·ind^−1^·h^−1^, which seems low given the higher clearance rates achieved by Cladocera. However, we note that these pollen contain substantially lower relative amounts of carbon, so that the Cladocera have to ingest relatively more zoospores to fulfill their nutritional needs. To our knowledge, no carbon ingestion rates have been published for *Keratella*. Other rotifer species such as *Brachionus rubens* and *Brachionus calycifloris* were shown to ingest up to 20–40 ng C·ind^−1^·h^−1^, which lies within the same order of magnitude (Rothhaupt 1990, 1995). Yet, although *Keratella* is smaller than *Brachionus*, its affinity for food is much higher (Walz 1993). As a consequence, with an equal food supply, *Keratella* is better at acquiring food, resulting in a higher ingestion rate.

The dynamical food‐chain model was parameterized with the results of the experiment (see Appendix S1: Fig. S1), and then used to estimate the minimum infection prevalence necessary to maintain zero rotifer population growth (i.e., the persistence of rotifer population at equilibrium). Thereafter, the model was used to estimate the minimum infection prevalence necessary for population persistence in other zooplankton groups with different body sizes. To this end, we applied species‐specific clearance rates and a simple allometric relationship between body size and biomass. Because of the limited availability of published data, we assumed that growth efficiency and mortality of zooplankton are identical for all zooplankton species, neglecting the dependence of these parameters on species and body size. For zooplankton with greater growth efficiency (which leads to a greater conversion factor *c*_*R*;_ see Appendix S1: Section S1) or lower natural mortality (*m*_*R*_), the minimum prevalence of infection will be underestimated (see Eqs. (6) and (7)). Also, if we consider more complex food webs in natural environments where there is additional mortality caused by higher trophic levels (greater *m*_*R*_), or greater resource subsidies from alternative resource (greater *g*_*B*_), the minimum prevalence of infection will be underestimated or overestimated, respectively. Our assumptions and analyses are aimed for understanding a general trend and variability of *P*_min_ depending on clearance rate and body size, but more details on species‐specific traits would be required to further improve the model.

Despite clear effects of rotifer grazing intensity on zoospore density, there was no observable knock‐on effect on infection prevalence. This is in line with earlier work that showed no effects of zooplankton grazing on phytoplankton prevalence of infection, but only on intensity of infection (Kagami et al. 2004). Our model explains this apparent lack of grazing effect through the minimum zoospore density necessary as food, in order to maintain the zooplankton population. More work is required, both experimentally and in the field, to include more realistic food‐web complexity than our host–parasite–grazer model system provides. Future studies may include other types of filter feeders, such as nonselective grazers that have higher clearance rates, for example, molluscs, sponges, or fish. However, these grazers may also consume larger‐celled phytoplankton, which are inedible to smaller zooplankton, leading to different phytoplankton population dynamics and thus also indirectly affecting spread of infection. Also, experiments could be performed that include an additional food source to sustain zooplankton growth. Natural phytoplankton communities consist of a mix of both inedible and edible species, where zoospores may provide an important complementary food source. Moreover, more edible phytoplankton species may support higher zooplankton densities, and with that increase overall zooplankton community clearance rate. In this case, rotifer population growth in itself might be fueled by grazing on other edible algae in the environment, but at the same time reduce the amount of zoospores derived from infections on larger inedible algae. Whether this will be sufficient to affect infection dynamics of the inedible phytoplankton species as well, and thereby its population densities, remains to be seen.

Our results indicate that even though fungal parasites comprise a high‐quality food source and can be grazed, this does not axiomatically result in a trophic cascade if their relation proves to be unsustainable in the longer run. However, we estimate that rotifers, and some other zooplankton groups, show a relatively high potential to reduce infections to a low level if their growth is supported, but also limited, by zoospores as a sole food source. This information may prove very valuable in the development of alternative biological strategies to control zoosporic diseases (Frenken et al. 2019). Because growth of zooplankton is food limited in our experiment setup, a logical following step would be to assess the potential of zooplankton for disease control in phytoplankton by adding natural complexity, such as additional food sources that would allow higher zooplankton population densities and thereby possibly also higher zoospore removal rates. Altogether, our findings highlight how complex three‐way interactions between hosts, parasites, and grazers can be, showing that trophic cascades are not always straightforward, and may depend on the nutritional demand of a grazer.

## Data Availability

Data are available from DataverseNL: https://hdl.handle.net/10411/JDNQTJ.
