# Supplementary material for: The potential of zooplankton in constraining chytrid epidemics in phytoplankton hosts
Source: Ecology. 2019 Oct 18;101(1):e02900. doi: 10.1002/ecy.2900 (PMC7003484; doi:10.1002/ecy.2900)
Supplement: Supplementary file 1 [file ECY-101-e02900-s001.pdf]

**Supporting Information.** Thijs Frenken, Takeshi Miki, Maiko Kagami, Dedmer B. Van de Waal, Ellen Van Donk, Thomas Rohrlack and Alena S. Gsell. 2019. The potential of zooplankton in constraining chytrid epidemics in phytoplankton hosts. *Ecology*.

Appendix S1

Section S1:

<u>Parameter estimation for Minimum prevalence of infection</u>

*Parameter estimation for this experiment*

In order to estimate the minimum prevalence of the infection $P_{min}$, we needed to estimate $m_R$, $m_R - g_B$, $c_R g_F$, $\beta$, and $m_A + 1/\tau$ . When both algae and chytrids are absent (K2), the rotifer follows a simpler exponential model ($dR/dt = - m_R R$) and when algae are present only (PK2), the rotifer follows another simpler exponential model ($dR/dt = g_B R - m_R R$). In order to estimate $m_R$, we fit the log solution log$R(t)$ = log$R(0) - m_R t$ linearly to the time series of K2 treatment. Similarly, we fit the log solution logR($t$) = log$R(0) - (m_R - g_B)t$ linearly to the time series of KP2 treatment. When chytrids were present ($F > 0$), the eqns. 2 and 4 are nonlinear differential equations and cannot be analytically solved so that we directly fit the eqns.2 and 4 to the experimental time series at each time point $t_j$ (= $\Delta j$ ) $t$using the approximated differential coefficients at $t_j$ as follows,

$$\left.\frac{dA_I}{dt}\right|_{t=t_j} \approx \frac{A_I(t_j + \Delta t) - A_I(t_j - \Delta t)}{2\Delta t},$$

$$\left.\frac{1}{R}\frac{dR}{dt}\right|_{t=t_j} \approx \frac{1}{R(t_j)}\frac{R(t_j+\Delta t)-R(t_j-\Delta t)}{2\Delta t},$$

where $j$ = 0, 1, 2,…7 and $\Delta t$ = 2 [day] in our datasets.

Since the eqns.2 and 4 are linear in terms of $\beta$, $m_A + 1/\tau$, and $c_{RgF}$ respectively, we can apply simple linear regressions to estimate these three parameters. Instead of pooling all of the time series data ($0 \le j \le 7$), we chose $j$ = 4, which realized the largest $R^2$ of the linear regression to estimate these parameters among $j$ that gave the estimates with reasonable sign ($\beta$, $m_A + 1/\tau$, and $c_{RgF} > 0$ ).

For $\left.\frac{dA_I}{dt}\right|_{t=t_j}$ and $\left.\frac{1}{R}\frac{dR}{dt}\right|_{t=t_j}$, the adjusted $R^2$ value was 0.5401 (P = 0.0003568) and 0.1903 (P = 0.04546), respectively. The relationship between the predicted values of $\left.\frac{dA_I}{dt}\right|_{t=t_j}$ (Prediction) with estimated values of $\beta$, $m_A + 1/\tau$ and the observed values (Observation), and that of $\left.\frac{1}{R}\frac{dR}{dt}\right|_{t=t_j}$ (Prediction) with estimated value of $c_{RgF}$ and the observed values (Observation) were as follows (Fig. S1).

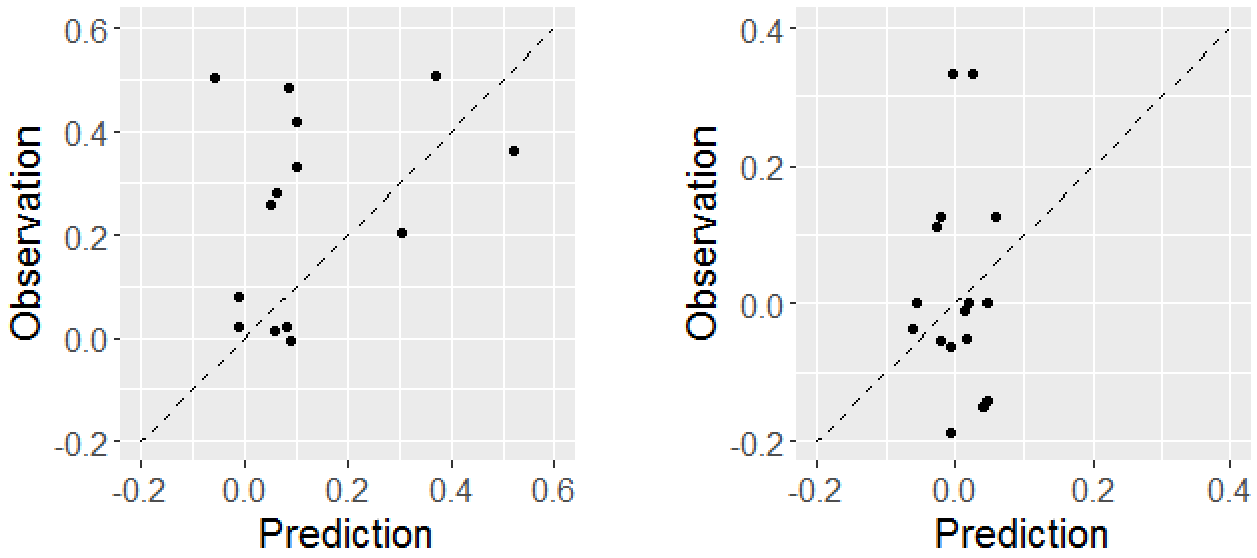


**Figure S1. The relationship between predicted and observed values.** The left panel represents the result for $\left.\frac{dA_I}{dt}\right|_{t=t_j}$ and the right panel represents the result for $\left.\frac{1}{R}\frac{dR}{dt}\right|_{t=t_j}$ at day 8.

*Parameter estimation from published data*

In order to estimate $c_{RgF}$ for other grazer species with different clearance rate, different body size, and different growth efficiency, we set additional assumptions as follows. First, we assumed that grazer growth is limited by carbon for simplicity, then the conversion factor consists of

three parameters: growth efficiency in terms of carbon ($e_R$), the carbon content of grazer ($q_R$) and the carbon content of zoospore ($q_F$). Therefore, we have

$$c_R g_F = e_R \frac{q_F g_F}{q_R}. \qquad \text{(S1)}$$

Although there would be nonlinear allometric relationship between carbon content and body size and also it would depend on taxonomy, we simply assumed that the carbon content is proportional to the cubic of body length of grazer ($l_R$) and zoospore ($l_F$). Then, we have

$$q_R = \alpha_R l_R^3, \text{ and } q_F = \alpha_F l_F^3, \qquad \text{(S2)}$$

where $a_R$ and $a_F$ are conversion coefficient from the body length to carbon content for grazer and zoospore, respectively. Finally, we assumed that the growth efficiency of grazer is independent of taxonomy and body size, due to the lack of information for many species. Then, setting $l_{R0}$, $l_{F0}$, and $g_{F0}$ as the body length of the grazer, that of zoospore, and clearance rate of the grazer from our experiments, eqn. S1 with eqn.S2 can be converted to the function of the grazer clearance rate $g_F$ and body length $l_R$ as follows,

$$
\begin{aligned}
c_R g_F &= e_R \frac{\alpha_F l_{F0}{}^3 g_F}{\alpha_R l_R{}^3} \\
&= e_R \frac{\alpha_F l_{F0}{}^3 g_{F0}}{\alpha_R l_{R0}{}^3} \left( \frac{g_F}{g_{F0}} \right) \left( \frac{l_{R0}}{l_R} \right)^3 \qquad \text{(S3)} \\
&= 1.561 \times 10^{-4} \left( \frac{g_F}{g_{F0}} \right) \left( \frac{l_{R0}}{l_R} \right)^3 \equiv c_R g_F (g_F, l_R).
\end{aligned}
$$

Although we did not measure the clearance rate of *Keratella* in this study,  we used the average value from several studies on the same species, i.e., 3.59 µl $ind^{-1}$ $h^{-1}$ (Table S1). We used our previous study for the grazer size, i.e., $l_{R0}$ = 110 um (Frenken et al. 2018, Table S1). Using these values with Eqn. S3, we estimated how the minimum prevalence of infection depends on species (Fig 5).

Table S1. Clearance rates and body lengths extracted from literature and used as input for calculating the minimum prevalence of infection as shown in fig 5 of the manuscript.

| Species | Group | Minimum clearance rate (µl $ind^{-1}$ $h^{-1}$) | Maximum clearance rate (µl $ind^{-1}$ $h^{-1}$) | Literature reference | Minimum body length (µm) | Maximum body length (µm) | Literature reference |
|---|---|---|---|---|---|---|---|
| *Keratella cochlearis* | Rotifer | 3.59 | 3.69 | This study, [1] | 110 | 111 | [1] |
| *Keratella cochlearis* | Rotifer | 0.65 | 8.13 | [2-12] | 72 | 140 | [1, 4, 6, 9, 13-15] |
| *Brachionus calyciflorus* | Rotifer | 1.45 | 30.6 | [16-22] | 175 | 213 | [15, 23, 24] |
| *Polyarthra dolichoptera* | Rotifer | 0.16 | 2.36 | [2, 10-12] | 140 | 220 | [11, 25] |
| *Polyarthra vulgaris* | Rotifer | 14.5 | 47 | [9, 13] | 110 | 120 | [9, 13, 26] |
| *Bosmina Longirostris* | Cladocera | 1.74 | 319 | [2, 6, 9, 10, 13, 27, 28] | 320 | 460 | [6, 9, 13, 29, 30] |
| *Daphnia magna* | Cladocera | 600 | 7250 | [6, 31-35] | 3330 | 4000 | [6, 30, 36] |
| *Daphnia pulex* | Cladocera | 1394 | 5667 | [6, 35, 37-39] | 1730 | 2000 | [6, 30, 36] |
| *Daphnia hyalina* | Cladocera | 787.5 | 900 | [6, 37, 39] | 1770 | 2380 | [6, 30, 40] |
| *Daphnia Pulicaria* | Cladocera | 1200 | 1890 | [37, 41, 42] | 1750 | 2800 | [30, 40, 43, 44] |
| *Diaptomus minutus* | Copepods | 790 | 804.9 | [9, 13] | 890 | 980 | [6, 9, 13, 30] |
| *Eudiaptomus gracilis* | Copepods | 194.8 | 1500 | [6, 39, 41, 45] | 835 | 1320 | [6, 30, 46] |
| *Cyclops abyssorum* | Copepods | 34.8 | 34.8 | [6] | 1400 | 1543 | [30, 47] |
| *Cyclops kolensis* | Copepods | 14.2 | 14.2 | [6] | 1120 | 1120 | [6] |
| *Strombidium* | Ciliates | 0.0897 | 4.7 | [48-50] | 22 | 104 | [25, 51] |
| *Halteria grandinella* | Ciliates | 0.0829 | 1.18 | [49, 52] | 20 | 25 | [6, 51] |
| *Coleps sp.* | Ciliates | 0.033 | 0.045 | [52] | 42 | 42 | [25] |
| *Urotricha spp.* | Ciliates | 0.005 | 0.078 | [52] | 25 | 25 | [4] |
| *Tintinnopsis* | Ciliates | 1.9 | 4.8 | [50] | 58 | 71 | [51] |
| *Balanion planctonicum* | Ciliates | 0.72 | 0.72 | [53] | 25 | 25 | [4] |

**List of references**

1. Frenken T, Wierenga J, Donk Ev, Declerck SAJ, Senerpont Domis LN, Rohrlack T, Van de Waal DB (2018) Fungal parasites of a toxic inedible cyanobacterium provide food to zooplankton. Limnology and Oceanography 63: 2384-2393. doi: 10.1002/lno.10945
2. Gilbert J, Bogdan K (1981) Selectivity of *Polyarthra* and *Keratella* for flagellate and aflagellate cells. Proceedings-International Association of Theoretical and Applied Limnology.
3. Telesh IV, Ooms-Wilms AL, Gulati RD (2006) Use of fluorescently labelled algae to measure the clearance rate of the rotifer *Keratella cochlearis*. Freshwater Biology 33: 349-355. doi: doi:10.1111/j.1365-2427.1995.tb00397.x
4. Weisse T, Frahm A (2002) Direct and indirect impact of two common rotifer species (*Keratella* spp.) on two abundant ciliate species (*Urotricha furcata*, *Balanion planctonicum*). Freshwater Biology 47: 53-64. doi: doi:10.1046/j.1365-2427.2002.00780.x
5. Walz N (1993) Carbon metabolism and population dynamics of *Brachionus angularis* and *Keratella cochlearis*Plankton Regulation Dynamics. Springer, pp. 89-105
6. Jürgens K, Wickham SA, Rothhaupt KO, Santer B (1996) Feeding rates of macro- and microzooplankton on heterotrophic nanoflagellates. Limnology and Oceanography 41: 1833-1839. doi: 10.4319/lo.1996.41.8.1833
7. Kirk KL, Gilbert JJ (1992) Variation in herbivore response to chemical defenses: zooplankton foraging on toxic cyanobacteria. Ecology 73: 2208-2217. doi: doi:10.2307/1941468
8. Gilbert JJ, Durand MW (1990) Effect of *Anabaena* flos ‐ aquae on the abilities of *Daphnia* and *Keratella* to feed and reproduce on unicellular algae. Freshwater Biology 24: 577-596.
9. Bogdan KG, Gilbert JJ (1987) Quantitative comparison of food niches in some freshwater zooplankton. Oecologia 72: 331-340. doi: 10.1007/BF00377560
10. Bogdan KG, Gilbert JJ (1982) Seasonal patterns of feeding by natural populations of *Keratella*, *Polyarthra*, and *Bosmina*: Clearance rates, selectivities, and contributions to community grazing. Limnology and Oceanography 27: 918-934. doi: doi:10.4319/lo.1982.27.5.0918
11. Bogdan KG, Gilbert JJ, Starkweather PL (1980) In situ clearance rates of planktonic rotifers. Hydrobiologia 73: 73-77. doi: 10.1007/BF00019428
12. Ronneberger D (1998) Uptake of latex beads as size-model for food of planktonic rotifers. Hydrobiologia 387: 445-449. doi: 10.1023/A:1017046711821
13. Bogdan KG, Gilbert JJ (1984) Body size and food size in freshwater zooplankton. Proceedings of the National Academy of Sciences 81: 6427-6431.
14. Stemberger RS, Gilbert JJ (1984) Spine development in the rotifer *Keratella cochlearis*: induction by cyclopoid copepods and *Asplanchna*. Freshwater Biology 14: 639-647. doi: 10.1111/j.1365-2427.1984.tb00183.x
15. Stemberger RS, Gilbert JJ (1985) Body size, food concentration, and population growth in planktonic rotifers. Ecology 66: 1151-1159. doi: 10.2307/1939167
16. Starkweather P, Gilbert JJ, Frost TM (1979) Bacterial feeding by the rotifer *Brachionus calyciflorus*: Clearance and ingestion rates, behavior and population dynamics. Oecologia 44: 26-30. doi: 10.1007/BF00346392
17. Starkweather PL, Gilbert JJ (1977) Feeding in the rotifer *Brachionus calyciflorus*. Oecologia 28: 133-139. doi: 10.1007/bf00345248

18. Rothhaupt K (1990) Differences in particle size-dependent feeding efficiencies of closely related rotifer species. Limnology and Oceanography 35: 16-23. doi: 10.4319/lo.1990.35.1.0016
19. Rothhaupt KO (1990) Changes of the functional responses of the rotifers *Brachionus rubens* and *Brachionus calyciflorus* with particle sizes. Limnology and Oceanography 35: 24-32.
20. Starkweather PL, Bogdan KG (1980) Detrital feeding in natural zooplankton communities: discrimination between live and dead algal foods. In: Dumont, HJ, Green, J (eds.) Rotatoria. Springer Netherlands, pp. 83-85
21. Mohr S, Adrian R (2000) Functional responses of the rotifers *Brachionus calyciflorus* and *Brachionus rubens* feeding on armored and unarmored ciliates. Limnology and Oceanography 45: 1175-1179. doi: doi:10.4319/lo.2000.45.5.1175
22. Gilbert JJ, Jack JD (1993) Rotifers as predators on small ciliates. In: Gilbert, JJ, Lubzens, E, Miracle, MR (eds.) Rotifer Symposium VI. Springer Netherlands, Dordrecht, pp. 247-253.
23. Nandini S (2000) Responses of rotifers and cladocerans to *Microcystis aeruginosa* (Cyanophyceae): A demographic study. Aquat Ecol 34: 227-242. doi: 10.1023/A:1009986928706
24. Xi Y-L, Liu G-Y, Jin H-J (2002) Population growth, body size, and egg size of two different strains of *Brachionus calyciflorus* Pallas (Rotifera) fed different algae. Journal of Freshwater Ecology 17: 185-190. doi: 10.1080/02705060.2002.9663886
25. Sarnelle O (1997) *Daphnia* effects on microzooplankton: comparisons of enclosure and whole-lake responses. Ecology 78: 913-928. doi: doi:10.1890/0012-9658(1997)078[0913:DEOMCO]2.0.CO;2
26. Virro T (1995) The genus *Polyarthra* in Lake Peipsi. In: Ejsmont-Karabin, J, Pontin, RM (eds.) Rotifera VII. Springer Netherlands, Dordrecht, pp. 351-357.
27. Tóth LG, Kato K (1997) Size-selective grazing of bacteria by *Bosmina longirostris*—an image-analysis study. Journal of Plankton Research 19: 1477-1493. doi: 10.1093/plankt/19.10.1477
28. DeMott WR (2003) Feeding selectivities and relative ingestion rates of *Daphnia* and *Bosmina*. Limnology and Oceanography 27: 518-527. doi: doi:10.4319/lo.1982.27.3.0518
29. Zaret TM, Kerfoot WC (1975) Fish predation on *Bosmina longirostris*: body-size selection versus visibility selection. Ecology 56: 232-237. doi: doi:10.2307/1935317
30. Hébert M-P, Beisner BE, Maranger R (2016) A meta-analysis of zooplankton functional traits influencing ecosystem function. Ecology 97: 1069-1080. doi: doi:10.1890/15-1084.1
31. Lürling M (2003) Effects of microcystin-free and microcystin-containing strains of the cyanobacterium *Microcystis aeruginosa* on growth of the grazer *Daphnia magna*. Environmental Toxicology 18: 202-210. doi: doi:10.1002/tox.10115
32. Hartgers EM, Heugens EHW, Deneer JW (1999) Effect of Lindane on the Clearance Rate of *Daphnia magna*. Archives of Environmental Contamination and Toxicology 36: 399-404. doi: 10.1007/PL00006612
33. Burns CW, Gilbert JJ (2003) Effects of daphnid size and density on interference between *Daphnia* and *Keratella cochlearis*. Limnology and Oceanography 31: 848-858. doi: doi:10.4319/lo.1986.31.4.0848

34. Soares MCS, Lürling M, Panosso R, Huszar V (2009) Effects of the cyanobacterium *Cylindrospermopsis raciborskii* on feeding and life-history characteristics of the grazer *Daphnia magna*. Ecotoxicology and Environmental Safety 72: 1183-1189.
35. Van Donk E, Lürling M, Hessen DO, Lokhorst GM (2003) Altered cell wall morphology in nutrient-deficient phytoplankton and its impact on grazers. Limnology and Oceanography 42: 357-364. doi: doi:10.4319/lo.1997.42.2.0357
36. Burns CW (2003) Relation between filtering rate, temperature, and body size in four speceis of *Daphnia*. Limnology and Oceanography 14: 693-700. doi: doi:10.4319/lo.1969.14.5.0693
37. DeMott WR, Zhang Q-X, Carmichael WW (1991) Effects of toxic cyanobacteria and purified toxins on the survival and feeding of a copepod and three species of *Daphnia*. Limnology and Oceanography 36: 1346-1357. doi: doi:10.4319/lo.1991.36.7.1346
38. Lürling M, Van Donk E (2003) Life history consequences for *Daphnia pulex* feeding on nutrient-limited phytoplankton. Freshwater Biology 38: 693-709. doi: doi:10.1046/j.1365-2427.1997.00242.x
39. Gulati RD (1989) Structure and feeding activities of zooplankton community in Lake Zwemlust, in the two years after biomanipulation. Hydrobiological Bulletin 23: 35-48. doi: 10.1007/BF02286425
40. Peter H, Lampert W (2003) The effect of *Daphnia* body size on filtering rate inhibition in the presence of a filamentous cyanobacterium. Limnology and Oceanography 34: 1084-1089. doi: doi:10.4319/lo.1989.34.6.1084
41. DeMott WR (1989) Optimal foraging theory as a predictor of chemically mediated food selection by suspension-feeding copepods. Limnology and Oceanography 34: 140-154. doi: doi:10.4319/lo.1989.34.1.0140
42. Infante A, Litt AH (2003) Differences between two species of *Daphnia* in the use of 10 species of algae in Lake Washington. Limnology and Oceanography 30: 1053-1059. doi: doi:10.4319/lo.1985.30.5.1053
43. Gliwicz ZM, Boavida MJ (1996) Clutch size and body size at first reproduction in Daphnia pulicaria at different levels of food and predation. Journal of Plankton Research 18: 863-880. doi: 10.1093/plankt/18.6.863
44. Jørgensen SE (1991) Handbook of ecological parameters and ecotoxicology. Elsevier
45. DeMott WR (1988) Discrimination between algae and artificial particles by freshwater and marine copepods. Limnology and Oceanography 33: 397-408. doi: doi:10.4319/lo.1988.33.3.0397
46. Svensson J-E (1997) Fish predation on *Eudiaptomus gracilis* in relation to clutch size, body size, and sex: a field experiment. Hydrobiologia 344: 155-161. doi: 10.1023/A:1002966614054
47. Ludovisi A, Todini C, Pandolfi P, Taticchi MI (2008) Scale patterns of diel distribution of the copepod *Cyclops abyssorum* Sars in a regulated lake: the relative importance of physical and biological factors. Journal of Plankton Research 30: 495-509. doi: 10.1093/plankt/fbn017
48. Børsheim KY (1984) Clearance rates of bacteria-sized particles by freshwater ciliates, measured with monodisperse fluorescent latex beads. Oecologia 63: 286-288. doi: 10.1007/BF00379891
49. Sanders RW, Porter KG, Bennett SJ, DeBiase AE (1989) Seasonal patterns of bacterivory by flagellates, ciliates, rotifers, and cladocerans in a freshwater planktonic community. Limnology and Oceanography 34: 673-687. doi: 10.4319/lo.1989.34.4.0673
50. Kivi K, Setälä O (1995) Simultaneous measurement of food particle selection and clearance rates of planktonic oligotrich ciliates (Ciliophora: Oligotrichina). Marine Ecology Progress Series 119: 125-137.

51. Xu H, Jiang Y, Zhang W, Zhu M, Al-Rasheid KAS, Warren A (2013) Annual variations in body-size spectra of planktonic ciliate communities and their relationships to environmental conditions: a case study in Jiaozhou Bay, northern China. Journal of the Marine Biological Association of the United Kingdom 93: 47-55. doi: 10.1017/S0025315412001075
52. Šimek K, Macek M, Pernthaler J, Straškrabová V, Psenner R (1996) Can freshwater planktonic ciliates survive on a diet of picoplankton? Journal of Plankton Research 18: 597-613. doi: 10.1093/plankt/18.4.597
53. Müller H, Schlegel A (1999) Responses of three freshwater planktonic ciliates with different feeding modes to cryptophyte and diatom prey. Aquat Microb Ecol 17: 49-60.
